# The newly identified migration inhibitory protein regulates the radial migration in the developing neocortex

**DOI:** 10.1038/srep05984

**Published:** 2014-08-07

**Authors:** Suxiang Zhang, Yoshitaka Kanemitsu, Masashi Fujitani, Toshihide Yamashita

**Affiliations:** 1 Department of Molecular Neuroscience, Graduate School of Medicine, Osaka University, 2-2 Yamadaoka, Suita, Osaka 565-0871, Japan; 2 JST, CREST, 5, Sanbancho, Chiyoda-ku, Tokyo 102-0075, Japan; 3 Molecular Research Center for Children's Mental Development, United Graduate School of Child Development, Osaka University, 2-2 Yamadaoka, Suita, Osaka 565-0872, Japan

## Abstract

Neuronal migration is a crucial process in the organization of the developing cerebral cortex. Although a number of positive regulatory mechanisms of radial migration have been identified, negative cell-autonomous mechanisms have yet to be fully described. Here we report a newly identified Migration Inhibitory Protein (MINP, formerly known as 2900011O08Rik) that negatively regulates radial migration. *MINP* mRNA was specifically detected in the central and peripheral nervous system, and especially enriched in the cerebral cortex. MINP immunoreactivity co-localized with the neuronal marker Tuj1 and was detected in the cytoplasm of post-mitotic neurons. To elucidate the function of MINP in the developing brain, we performed *in utero* electroporation of *MINP* siRNA, *MINP* shRNA, or *MINP*-overexpressing vectors into mouse cortices and carried out *in vivo* migration assays. Whereas knockdown of MINP did not alter neuronal morphology, the radial migration was found accelerated by *MINP* knockdown, and reduced by *MINP* overexpression. This migration phenotype was also confirmed *in vitro*, indicating that MINP regulates neuronal migration in a cell-autonomous fashion. Furthermore, downregulation of MINP affected microtubule stability by interacting with tubulin that is a potential mechanism involved in the regulation of neuronal migration.

Neuronal migration is a crucial process in the organization of the developing cerebral cortex, as it regulates the appropriate formation of neuronal circuits^1^. Precise spatial and temporal coordination of neuronal migration is essential for the correct positioning and subsequent wiring of neurons into functional circuits. Impairments in migration result in structural defects leading to severe mental abnormalities, such as Miller-Dieker syndrome, which is associated with loss of Lissencephaly-1 (Lis1)^2^ and X-linked subcortical band heterotopia, which is related to compromised Doublecortin function^3^.

Genome-wide studies of copy number variations (CNVs) have provided clues about the etiology of neuropsychiatric disorders. Emerging evidence indicates that rare mutations confer significant risk in common neurological diseases^4^. Rearrangement at chromosome 16p13.11 is one of the well-characterized loci, which displays phenotypic variability. Psychiatric diagnoses of individuals carrying deletions in 16p13.11 have been associated with epilepsy^5^, mental retardation^6^ and schizophrenia^7^, while duplications in this region have been implicated in autism spectrum disorders^4^ as well as in attention deficit hyperactivity disorder (ADHD)^8^. These reports indicate that changes in the function of genes located in the 16p13.11 region are associated with psychiatric disorders. Within 16p13.11, Nuclear distribution gene E homologue 1 (NDE1) has been reported to be highly expressed in the developing cerebral cortex in cortical precursors and young post-mitotic neurons. NDE1 knockout mice display reduced cortical layers resulting in a smaller cerebral cortex^9^. This report implicates that NDE1 is a candidate gene for neuropsychiatric disorders associated with 16p13.11 CNV. However, whereas patients with a homozygous mutation in NDE1 exhibit severe migration deficits, heterozygous mutations of NDE1 in parents have no psychiatric phenotype^10^, indicating that there may be other candidate genes within the 16p13.11 region.

Here, we report a role of another susceptible gene 2900011O08Rik, which is also located on the 16p13.11 locus. This gene was specifically expressed in the nervous system and is highly expressed in post-mitotic neurons of the cerebral cortex. Moreover, *in vivo* downregulation of this gene specifically accelerated radial migration without changing the morphology and laminar organization of migrating neurons. We named this new gene *MINP* (Migration Inhibitory Protein) according to its distinct role in the developing cortex.

## Results

### *MINP* mRNA is expressed in the central and peripheral nervous system

**Figure 1:**
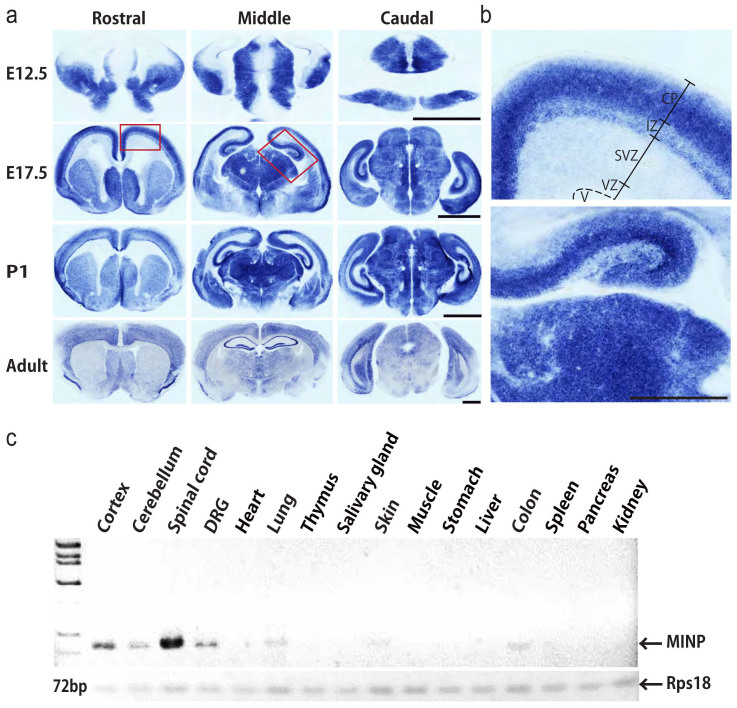
*MINP* mRNA expression in the central and peripheral nervous system. (a) Expression of *MINP* mRNA was analyzed by *in situ* hybridization on coronal sections of the rostral, middle, and caudal parts of E12.5, E17.5, P1, and adult mouse brain. Scale bars, 500 μm. (b) High magnification micrographs of E17.5 developing the cortex and hippocampus. CP, cortical plate; IZ, intermediate zone; SVZ, subventricular zone; VZ, ventricular zone; V, ventricle. Scale bar, 250 μm. (c) RT-PCR analysis for *MINP* mRNA in various tissues of the adult mouse. Rps18 was used as an internal control.

We first examined *in silico* the distribution of *MINP* mRNA in E14.5 mouse brain on transcriptome atlas database, Eurexpress^11^ A MINP riboprobe corresponding to the full-length cDNA of *MINP* detected expression in both the central and peripheral nervous system of E14.5 embryos. Based on this finding, we then investigated the developmental trajectory of *MINP* mRNA expression in the mouse brain. We obtained coronal sections through three different brain regions (rostral, middle and caudal) at embryonic day 12.5, 17.5 (E12.5, E17.5), postnatal day 1 (P1), and in adulthood, and performed *in situ* hybridization using the same riboprobe. As illustrated in Figure 1a, *MINP* mRNA could be detected more strongly in the ganglionic eminence and thalamus than in the preplate and ventricular zone at E12.5. At E17.5 and P1, MINP expression increased markedly in the cortical plate and hippocampus, and continued to be highly expressed in the thalamus and striatum. In adulthood, however, MINP was mainly concentrated in the cerebral cortex as well as the hippocampus, but was barely detectable in the striatum (Figure 1a). Examination of E17.5 labeling in the neocortex at higher magnification revealed that, MINP was specifically distributed in the intermediate zone (IZ) and the cortical plate (CP) while almost no signal was detectable in the proliferative ventricular zone (VZ) or the subventricular zone (SVZ) (Figure 1b). This pattern of distribution suggests that MINP is expressed in post-mitotic neurons, but not in precursor cells.

To determine whether MINP is also expressed in other organs, we performed RT-PCR on tissues dissected from adult mice. In contrast to robust signals in spinal cord, cerebral cortex, cerebellum and dorsal root ganglia (DRG), only negligible signals were detected in lung, skin, and colon (Figure 1c), demonstrating that MINP is discretely expressed in the central and peripheral nervous system in adult mouse as well as in the E14.5 embryo.

### MINP protein is expressed in post-mitotic cortical neurons

**Figure 2:**
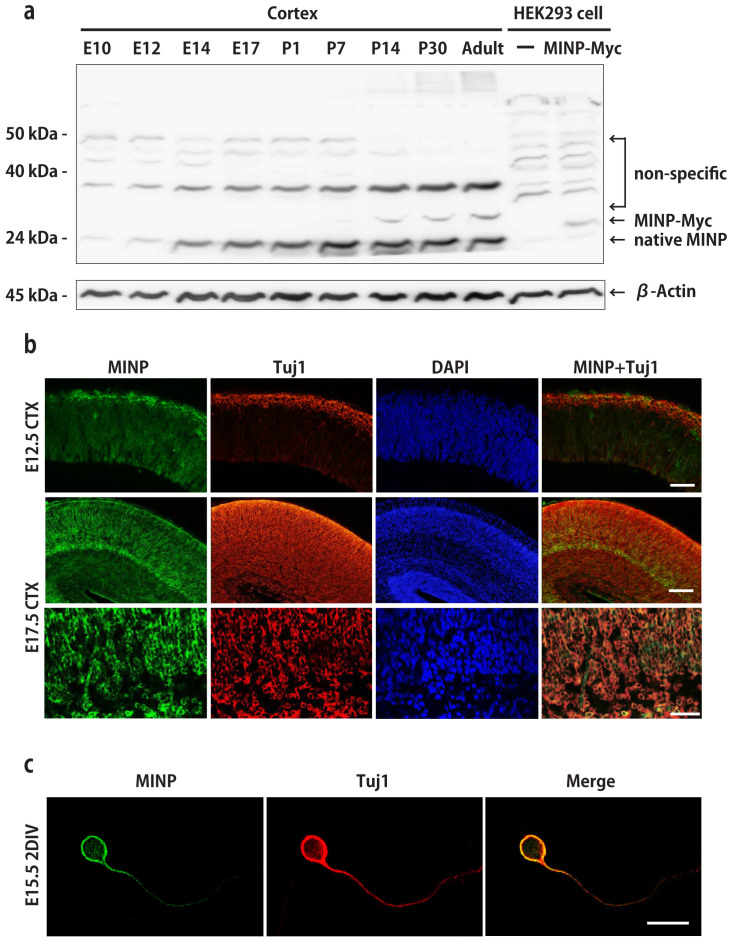
MINP protein expression in post-mitotic neurons. (a) Immunoblotting for MINP in mouse brain lysates at the indicated ages (lower arrow). The upper bands correspond to non-specific bands. β-Actin was used as a loading control. HEK293 cells transfected with an MINP-Myc-expressing vector was used as a positive control (middle arrow). (b) Immunostaining with MINP (green), Tuj1 (red) and DAPI (blue) in coronal sections of the E12.5 (top panels) and E17.5 (second panels) mouse brain. The third panels show co-localization of MINP (green) and Tuj1 (red) in the intermediate zone and cortical plate of the E17.5 mouse brain, as analyzed by confocal microscopy. Scale bars, 50 μm, 20 μm (third panel). (c) Subcellular localization of MINP was examined by confocal microscopy in a cortical neuron immunostained with MINP (green), Tuj1 (red) and DAPI (blue). Scale bar, 10 μm.

Next, we investigated the expression of MINP protein. We generated a rabbit polyclonal antibody against MINP and evaluated the specificity of the MINP antibody. Cortical lysates from E10, E12, E14, E17, P1, P7, P14, P30, and adult mouse were analyzed with purified anti-MINP antibody by Western blot analysis. An immunoreactive band was detected at 24 kDa, consistent with the predicted molecular weight of MINP. Several additional bands were detected around 40 kDa and 50 kDa, which were presumably non-specific (Figure 2a). To confirm that the band detected at 24 kDa represents the MINP protein, cell lysates of HEK293 cells transfected with pCAG-MINP-Myc expressing vectors were used as a positive control (Figure 2a, arrow). We found that only a faint signal was observed at E10, a developmental stage during which cortical precursors, but not neurons, occupy the main cell population in the neocortex. MINP expression gradually increased during cortical neurogenesis (E12, E14, and E17), peaked at P7, and this level of expression was maintained until adulthood. Hence, the MINP protein is expressed in mouse cortices of all ages and the expression level may be linked to the number of neurons at the corresponding ages.

To confirm that MINP is expressed in neurons as indicated by *in situ* hybridization analysis, we carried out immunohistochemical double-labeling experiments in developing mouse brains with anti-MINP antibody and Tuj1 (anti-βIII tubulin antibody), a neuronal marker. At E12.5, MINP signal was restricted to the preplate of the cortex, which was also marked by Tuj1, whereas no signals could be observed in the ventricular (VZ) or subventricular zone (SVZ) (Figure 2b, top panels). At E17.5, robust MINP protein expression was detected in the cortical plate (CP) as well as the intermediate zone (IZ) of the developing cortex but was barely detected in the ventricular zone (VZ) and the subventricular zone (SVZ) (Figure 2b, second panels), which is consistent with the findings by *in situ* hybridization (Figure 1b). Confocal micrographs from IZ and CP showed that most of MINP-positive cells overlapped with Tuj1-positive neurons (Figure 2b, third panels), indicating that MINP is expressed in post-mitotic neurons.

Next, we assessed the subcellular localization of MINP in cortical neurons by examining the distribution of immunoreactivity using confocal microscopy. Cultured E15.5 cortical neurons were immunostained with anti-MINP antibody and Tuj1 at 2 DIV. MINP was co-localized with βIII tubulin and distributed surrounding the nucleus (Figure 2c), indicating that MINP protein localizes to the cytoplasm. To observe whether MINP was also expressed in other subcellular organelles, we double stained for MINP and several organelle-specific proteins (Supplementary figure 1). Although MINP signals were partially overlapped with the Transferrin Receptor (TfnR) (a marker for recycling endosomes) and Syntaxin6 (a marker for *trans*-Golgi network), these co-localizations were not as significant as the co-localization with Tuj1. These observations indicate that MINP is highly associated with tubulin and microtubules.

### MINP negatively regulates radial migration

**Figure 3:**
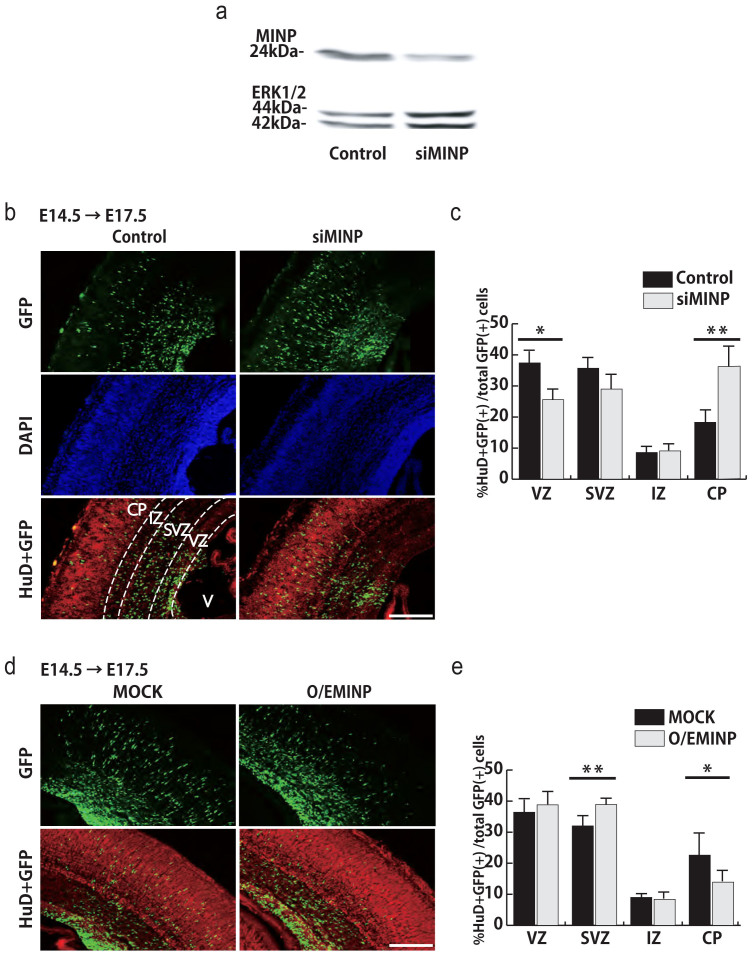
Acute knockdown of *MINP* accelerates radial migration, while *MINP* overexpression suppresses it. (a) Western blots used for quantifying the knockdown efficiency of *MINP* siRNA in E14.5 cultured cortical neurons. ERK1/2 was used as a loading control. (b) Control or *MINP* siRNA in combination with nuclear GFP was co-electroporated into E14.5 cortices. Three days after electroporation, coronal sections were immunostained for nGFP (green), HuD (red) and DAPI (blue). Scale bar, 100 μm. (c) Quantification of electroporated GFP and HuD double-positive cells in each cortical layer. CP, cortical plate; IZ, intermediate zone; SVZ, subventricular zone; VZ, ventricular zone; V, ventricle (mean ± SEM; n = 5, each group). *p < 0.01, **p < 0.005, Student's *t*-tests. (d) Immunostaining of control (MOCK) or MINP-overexpressing (O/EMINP) cortices at E17.5. *In utero* electroporation was carried out the same way as MINP knockdown. Scale bar, 100 μm. (e) Quantification of cells in each cortical layer shows that, compared with the control, more MINP-overexpressing neurons migrated into the cortical plate (mean ± SEM; n = 4, each group). *p < 0.01, **p < 0.005, Student's *t*-tests.

To elucidate the function of MINP in the developing cortex, we performed a gain-of-function experiment in cortical precursors by ectopic expression of MINP. A Carboxy-terminally Myc-tagged MINP expressing vector was generated and confirmed its expression in HEK293 cells (Supplementary figure 2a). We first overexpressed MINP in E12.5 precursors *in vitro* and examined whether MINP was involved in neurogenesis by immunostaining for the cell proliferation marker Ki67 at 2 DIV as well as with the neuronal differentiation marker Tuj1 at 3 DIV. The results showed a similar percentage of Ki67-positive and Tuj1-positive cells between control (MOCK) and MINP-overexpressing cells (O/EMINP) (Supplementary figure 2b and c), indicating that ectopic expression of MINP had no effect on proliferation or differentiation of cortical precursor cells *in vitro*. We then carried out a loss-of-function experiment using *MINP*-specific siRNA. First, we evaluated the knockdown efficiency of *MINP* siRNA in E14.5 cortical neurons. siRNA nucleofection of cortical neurons yielded almost 100% transfection efficiency as described previously^12^. Western blot analysis showed that nucleofection of *MINP* siRNA significantly suppressed MINP expression by 70% (Figure 3a). To determine whether endogenous MINP regulates cortical precursor cell proliferation and differentiation, we performed *in utero* electroporation of a nuclear GFP expressing vector in combination with either control or *MINP* siRNA into E14.5 cortices. Next, we carried out immunostaining for Ki67 at E16.5 or for neuronal maker HuD at E17.5 (Supplementary figure 2d and f). The proportion of GFP-labeled Ki67-positive proliferating cells and HuD-positive differentiated neurons was not changed by *MINP* knockdown relative to controls (Supplementary figure 2e and g). These results indicate that neither neural precursor cell proliferation nor differentiation was affected by MINP.

We then asked if MINP was associated with radial migration. *In utero* electroporation into E14.5 cortices was performed with a nuclear GFP expressing vector in combination with control or *MINP* siRNA, followed by immunocytochemical labeling for GFP, HuD and DAPI at E17.5 (Figure 3b). We counted GFP and HuD double-labeled neurons in each cortical layer. As shown in Figure 3c, in the cortical plate, the number of *MINP* siRNA-transfected neurons increased by approximately two fold compared with control-transfected neurons. Conversely, fewer *MINP* siRNA-transfected cells remained in the VZ compared with control-transfected cells, suggesting that *MINP* knockdown accelerated the radial migration of cortical neurons.

To determine whether radial migration would be delayed by *MINP* overexpression, we co-electroporated a nuclear GFP-expressing vector combined with either control or *MINP*-expressing vectors into E14.5 cortices, and immunostained for GFP and HuD at E17.5 (Figure 3d). The percentage of GFP and HuD double-positive neurons in the CP was significantly reduced by ectopic expression of MINP (Figure 3e). Indeed, we found that more *MINP*-overexpressing cells had accumulated in the SVZ, indicating that *MINP* overexpression had suppressed radial migration. These results strongly suggest that MINP acts as a negative factor of radial migration.

### Knockdown of MINP delays the radial migration of cortical neurons but does not affect their morphology, final layer positioning and differentiation in the cerebral cortex

**Figure 4:**
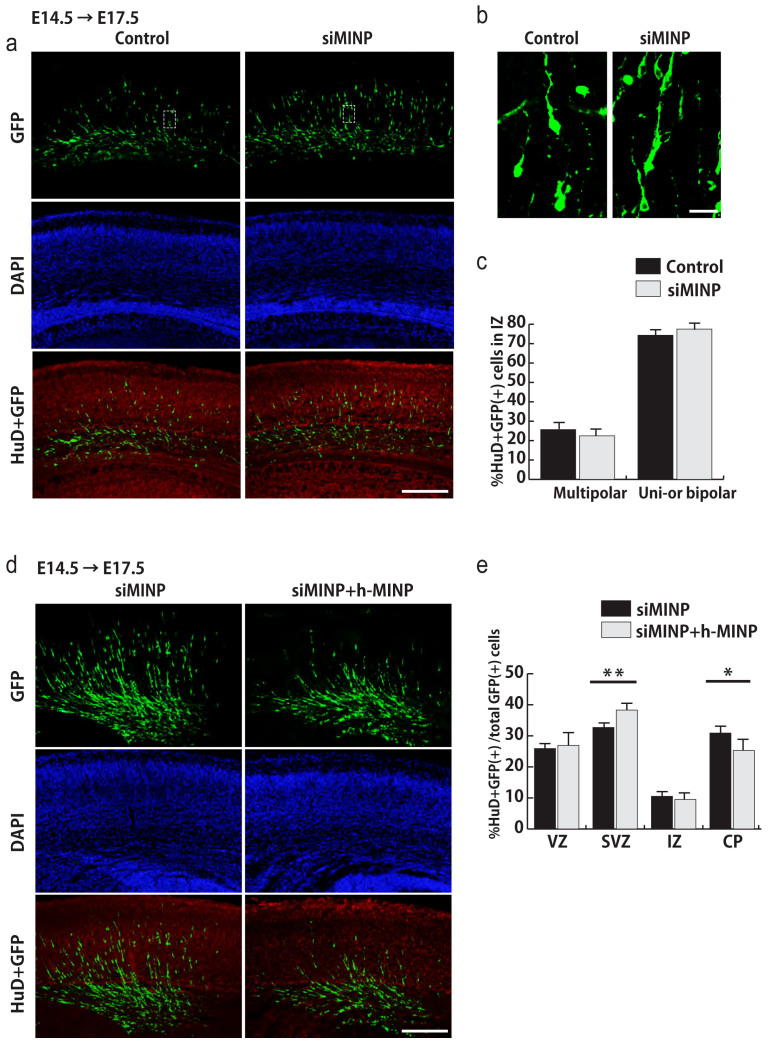
Neuronal morphology is not perturbed by knockdown of *MINP* at intermediate zone. Affected radial migration is successfully rescued by ectopic expression of siRNA resistant human-MINP. (a) Control or *MINP* siRNA in combination with pCAG-EGFP was electroporated at E14.5 and immunostained at E17.5 for GFP (green), HuD (red) and DAPI (blue). Scale bar, 100 μm. (b) Confocal micrographs showing morphology of neurons expressing control or *MINP* siRNA. Scale bar, 20 μm. (c) The proportion of multipolar and uni-or bipolar neurons in the intermediate zone of cortices electroporated with control or *MINP* siRNA (mean ± SEM; n = 4, each group). It shows no significant differences between the two groups. (d) Micrographs of E17.5 cortices electroporated with *MINP* siRNA plus indicated expressing vectors at E14.5. Scale bar, 100 μm. (e) Quantification of electroporated GFP and HuD double-positive cells in each layer. The result shows that overexpression of human-MINP effectively suppressed the radial migration which accelerated by *MINP* siRNA (mean ± SEM; n = 4, each group). *p < 0.05, **p < 0.01, Student's *t*-tests.

It has been reported that migrating neurons display several morphological changes in the intermediate zone (IZ)^13^. Therefore, we analyzed whether acute loss of MINP leads to abnormal morphologies in these neurons. We electroporated E14.5 cortices with control or *MINP* siRNA plus pCAG-IRES-GFP expressing vector and immunostained these electroporated cells for GFP, HuD and DAPI at E17.5 (Figure 4a). We then counted GFP and HuD double-positive neurons exhibiting multipolar or uni-or bipolar morphologies in the IZ (Figure 4b). The quantification result showed that the proportion of multipolar and uni-or bipolar cells in IZ was not significantly changed in *MINP* siRNA-electroporated cortices, compared with control cortices (Figure 4c).

Next, to study whether prolonged knockdown of *MINP* leads to eventual migration defects at later developmental stage, we generated an *MINP* shRNA construct. We first nucleofected this construct into E14.5 cortical neurons to ensure a long-term effect. Cell lysates were harvested at 5 days as well as 7 days after nucleofection, followed by immunoblotting for MINP. MINP protein expression was decreased in *MINP* shRNA-nucleofected cortical neurons to approximately 35% at 5 DIV and 70% at 7 DIV (Supplementary figure 3a). We then co-electroporated nuclear GFP expressing vector in combination with either scrambled (Scr) or *MINP* shRNA into E14.5 cortices, and immunostained for GFP, DAPI and either Satb2 (a marker for upper layer neurons) or Ctip2 (a marker for layer V neurons) at P2. We observed no abnormalities in the cortical layers, nor were there any apparent mismigration by neurons in the cortical plate after *MINP* shRNA electroporation (Supplementary figure 3b). Most electroporated cells correctly migrated to the upper layer (II–IV) and quantification of the GFP positive cells also revealed no significant difference between scrambled and *MINP* shRNA-transfected cortices (Supplementary figure 3c). These results indicate that knockdown of MINP delays the radial migration of cortical neurons but does not affect their morphology, final layer positioning and differentiation in the cerebral cortex.

### Affected radial migration is successfully rescued by human-*MINP*

To solidify the result that *MINP* negatively regulates radial migration, we performed rescue experiments using pCAG-human-*MINP*-Myc-IRES-GFP expressing vector produced from cDNA of human-*MINP* (known as C16orf45), which conserves 97.1% amino acid sequence with mouse-*MINP* but not targeted by mouse *MINP* siRNA. After confirming protein expression by western blotting (Supplementary figure 2h), we electroporated this construct with MINP siRNA or pCAG-IRES-GFP vector into the cortices at E14.5 and analyzed the cortices at E17.5 (Figure 4d). We found that the electroporated cells co-expressing GFP in the cortical plate were significantly decreased in the rescue group (siMINP + h-MINP) compared with *MINP* siRNA-electroporated group (Figure 4e). In contrast, more GFP-positive cells accumulated in the subventricular zone in rescued cortices, displaying the similar phenotype observed in mouse-*MINP* overexpression (Figure 3e). Therefore, we excluded the possible off-target effect of the siRNA.

### *MINP* knockdown increases neural migration *in vitro*

**Figure 5:**
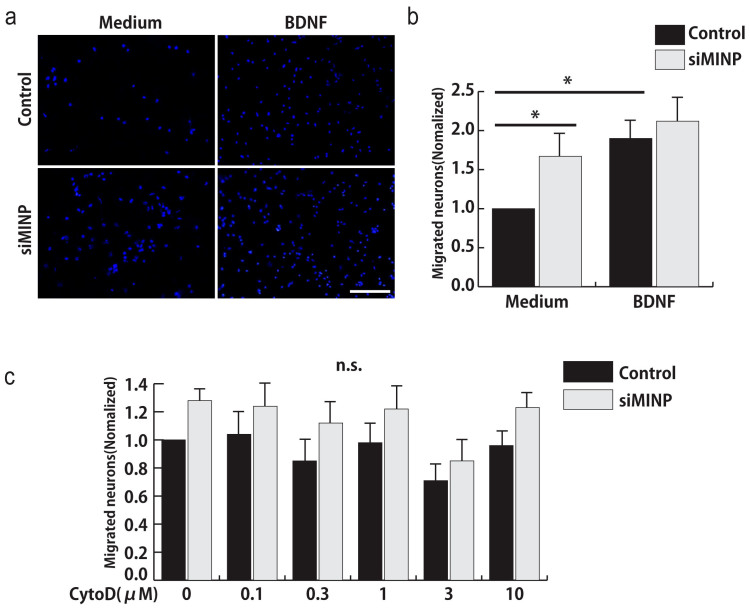
Knockdown of *MINP* increases the number of migrated neurons *in vitro*. (a) Images of migrated neurons immunostained for DAPI after a 16-h incubation in the Boyden chamber. Scale bar, 20 μm. (b) The graph represents the mean ± SEM of the number of migrated neurons normalized to the value of the control (n = 5, each group). *p < 0.05, one-way ANOVA followed by Tukey–Kramer tests. (c) Compared to the control, in *MINP* siRNA-transfected neurons, migration was not affected by treatment with CytoD, an inhibitor of *actin* dynamics (mean ± SEM; n = 4, each group).

In order to explain the relationship between MINP and neuronal migration, we performed an *in vitro* cell migration assay using MINP-depleted neurons. For this, we first isolated E14.5 cortical neurons, and then performed nucleofection with control or *MINP* siRNA. Two days after nucleofection, we observed almost 100% cells are post-mitotic cortical neurons (data not shown). Then, neurons were detached and replated on the upper well of the Boyden transwell chamber. After an additional incubation for 16 h, we performed DAPI staining on the bottom side of the membrane. For assessing competency, we added a chemo-attractive BDNF to the bottom well as a positive control^14^. Quantification of migrated neurons showed that the number of *MINP* siRNA-transfected neurons that moved through the transwell was 1.7 times more than that of the control-transfected neurons (Figure 5a and b), thereby supporting our results that *MINP* knockdown increased the neuronal migration *in vivo*. Moreover, in the presence of BDNF, the difference between the groups disappeared, suggesting that both control and MINP-depleted neurons showed maximum migratory activities because of the strong effects of BDNF.

The result of the transwell assay also indicated that MINP regulates neuronal migration in a cell-autonomous manner. Thus, we supposed that MINP is associated with modulating cytoskeletal structures or the motilities of neurons. Dynamics of both actin cytoskeleton and microtubules play a key role in regulating physical behaviors of migrating neurons^15^,^16^. To determine if perturbation of the actin assembly-disassembly system affects MINP-mediated neuronal migration, we first performed a cell migration assay using cytochalasin D (CytoD), a widely used inhibitor of actin polymerization. Control and *MINP* siRNA-tansfected neurons were incubated with different concentrations of CytoD, and quantified 16 h later. As shown in Figure 5c, the accelerated migration induced by *MINP* knockdown was not apparently affected by CytoD treatment, thereby excluding the possibility that MINP regulates the actin assembly-disassembly system.

### *MINP* expression influences microtubule stability by interacting with tubulin

We observed that MINP co-localized with βIII tubulin, an element that constitutes microtubules. Microtubules are the largest cytoskeletal components involved in various cellular events including mitosis, intracellular transport, as well as cell motility. During neural migration, leading process extension is followed by cell soma movement^16^. Notably, the whole process of neuronal migration is precisely regulated by tubulin polymerization-depolymerization cycles^17^. Thus, we presumed that MINP is a potential regulator of microtubule dynamic stability.

**Figure 6:**
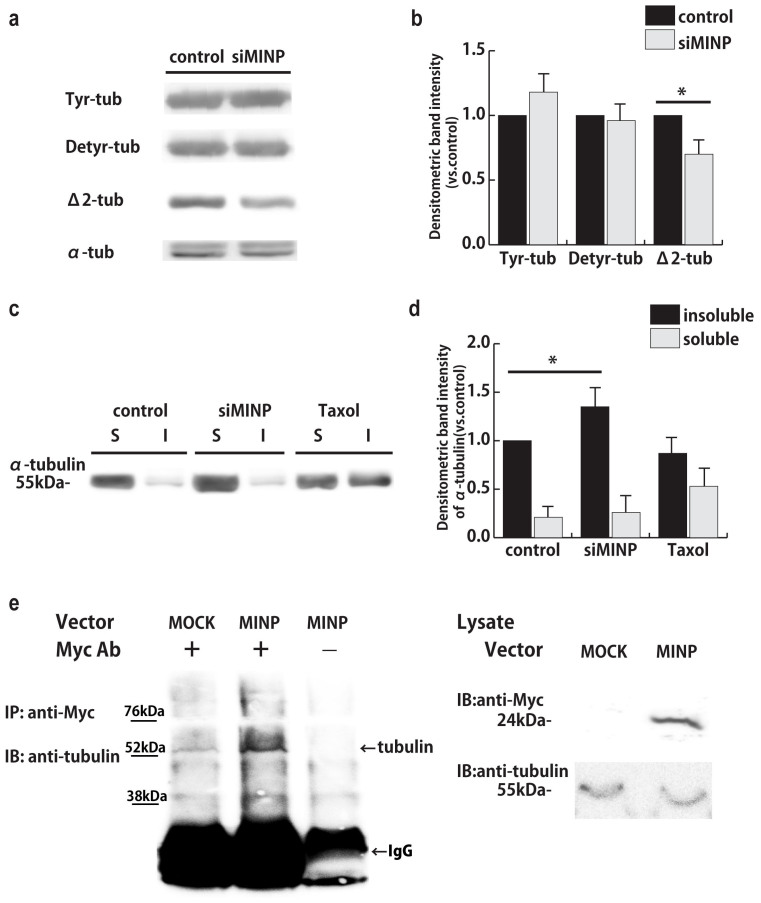
MINP regulates microtubule stability by interacting with tubulin. (a) Western blots analysis with the indicated tubulin antibodies. Cell lysates were obtained from cortical neurons nucleofected with control or *MINP* siRNA. α-tubulin was used as a loading control. (b) Histogram showing the relative intensity of the bands normalized to the value of the control in the western blots shown in (a) (mean ± SEM; n = 5, each group). *p < 0.05, Student's *t*-tests. (c) TX100-soluble (S) and insoluble (I) fractions were immunoblotted for α-tubulin. The insoluble fraction represents stable tubulin components, while the soluble fraction represents unstable tubulin. The microtubule stabilizer Taxol was used as positive control. (d) The relative intensity of the bands normalized to the value of the control in the immunoblots shown in (c) (mean ± SEM; n = 4, each group. *p < 0.05, one-way ANOVA followed by Tukey–Kramer tests). (e) Co-immunoprecipitation (IP) assay and immunoblot (IB) analyses of cortical neurons transfected with control (MOCK) or MINP-Myc overexpressing vector (MINP). Cell lysates were immunoprecipitated with anti-myc antibody and immunoblotted with α-tubulin. Lysate immunoprecipitated without Myc-antibody was used as negative control.

Recent studies have shown that post-translational modifications crucially control microtubule functions as well as dynamics^18^. We therefore examined tyrosinated tubulin (Tyr-tubulin), detyrosinated tubulin (Detyr-tubulin), and Δ2-tubulin, the three major tubulin subpopulations existing in neuronal cells^19^. E14.5 cortical neurons were transfected with control or *MINP* siRNA and cultured for 3 DIV. We then harvested cell lysates and then performed immunoblotting against Tyr-tubulin, Detyr-tubulin, and Δ2-tubulin as well as α-tubulin. While no significant changes could be observed in Tyr-tubulin or Detyr-tubulin pools, Δ2-tubulin was significantly decreased by approximately 30% in MINP-suppressed neurons (Figure 6a and b). Given that Δ2-tubulin represents a stable tubulin assembly, the result suggested that microtubule stability is affected by the downregulation of MINP.

Next, to determine whether unstable tubulin components are increased in the MINP-depleted cell lysate, we performed a tubulin assay. Control or *MINP* siRNA-transfected neurons were harvested in PEM-Triton X buffer, and then separated into Triton-X soluble and insoluble tubulin fractions, which represent the unstable and stable tubulin, respectively^20^. We used a Taxol-treated sample as a positive control, and performed immunoblotting against α-tubulin. Quantification showed that the soluble tubulin fraction derived from the MINP-suppressed cell lysate was significantly increased by approximately 35% compared to the similar fraction derived from the control (Figure 6c and d). This finding indicated that *MINP* knockdown decreased microtubule stability by increasing the proportion of unstable tubulin.

Finally, to better understand how MINP regulates microtubule, we carried out co-immunoprecipitation experiments. E14.5 cortical neurons were nucleofected with control (MOCK) or pCAG-MINP-Myc-expressing vector (MINP) and cultured for additional 2 days *in vitro*. Cell lysates were immunoprecipitated with anti-Myc antibody and immunoblotted with anti-α-tubulin antibody. Lysate without applying Myc antibody was used as negative control. As shown in Figure 6e, more endogenous tubulin was detected in *MINP* overexpressed-cell lysate, indicating that MINP can interact with tubulin either directly or indirectly.

This result strongly supports our notion that MINP regulates the neuronal migration at least by modulating the stability of microtubule dynamics that resulted from interaction between tubulin and MINP protein.

## Discussion

In this study, we identified the role of a novel gene *MINP* in the developing neocortex. Owing to its distinct chromosomal location, *MINP* is considered a candidate gene for 16p13.11-related neurodevelopmental disorders. We found that MINP protein is highly expressed in the cerebral cortex, especially in post-mitotic cortical neurons. Acute knockdown of MINP accelerates the radial migration of cortical neurons; however, no significant abnormalities are observed in neuronal morphology and cortical lamination. Finally, MINP interacts with tubulin and the downregulation of MINP affects microtubule stability that could lead to the alteration of neuronal migration.

Our findings illustrated that MINP acts as a negative regulator of radial migration. During radial migration, projection neurons encounter several extracellular guidance cues such as semaphorins and reelin that aid in maintaining the migratory state^21^. These extracellular factors act through intracellular signaling cascades, which in turn initiate diverse positive and negative effects on cell locomotion by regulating movement, modulating speed, and influencing navigation^21^. Although there are numerous reports on positive regulatory mechanisms of radial migration, the negative regulatory mechanisms have remained unanswered. However, recent studies have demonstrated that cortical neurons also respond to negative cues that impede radial migration^20^,^22^, thereby providing new evidence that the coordinative contribution of negative factors is also necessary for radial migration.

If this were the case, then how does MINP work as a negative regulator? We suggest that MINP regulates neuronal migration in a cell-autonomous manner. Given that MINP protein localizes in the cytoplasm and interacts with tubulin, MINP has been engaged in the intracellular signaling pathway to regulate tubulin cytoskeleton. Previous studies have shown that migrating neurons exhibit multiple steps including the early phase of the multipolar migration mode, bipolar locomotion mode, and the final radial glia-independent translocation mode^23^,^24^,^25^. According to our data, MINP is mainly distributed in the neurons of the intermediated zone (IZ) and cortical plate (CP), in which most of the migrating neurons transformed into the locomotion mode and displayed bipolar morphology with a leading process^26^. We also found that acute knockdown of MINP affects neither multipolar stage exit nor neuronal polarity *in vivo*. Taken together, these findings suggest that MINP may specifically regulate the migration rate of locomoting neurons.

A recent study showed that treatment with inhibitors for cyclin-dependent kinase 5 (Cdk5), Src family kinase, and c-Jun N-terminal kinase (JNK) suppressed the migration rate of the locomoting neurons in cortical slice tissues^26^. However, the precise molecular mechanism underlying this phenomenon has not yet been fully understood. One possibility is that MINP may directly interact with these kinases and regulate their downstream signals such as DCX or MAP1B^25^,^26^. Because our data show that MINP is involved in regulating microtubule stability by interacting with tubulin, which raises another possibility that MINP act together with microtubule regulatory proteins, and finally contribute to tubulin assembly and microtubule dynamics.

Our data also revealed that downregulation of MINP decreases Δ2-tubulin. Previous studies have shown that Δ2-tubulin is only detectable in the brain, especially in the axons and dendrites of differentiated neurons^19^,^27^. In addition, Δ2-tubulin is absent in early-born neurons and glial cells, and its expression is similar to that of MINP. As a product of tubulin post-translational modifications, Δ2-tubulin is generated from detyrosinated tubulin via an irreversible process, which exits from tubulin tyrosination-detyrosination cycle^17^. Therefore, MINP may be involved in the production of Δ2-tubulin without affecting the tyrosinated and detyrosinated tubulin pools. Since the underlying enzyme mediating this reaction has not yet been identified, the relationship between MINP and Δ2-tubulin remains an unsolved issue that should be addressed in future studies.

Moreover, studies have demonstrated that modulating neuronal migration also depends on specific neurotransmitter receptors and voltage-dependent Ca^2+^ channels as well as Rab11-dependent recycling pathways; these provide new intracellular trafficking signals that play crucial roles in regulating the neuronal migration^13^,^28^,^29^. Our data suggest that MINP is partially co-localized with recycling endosomes and Golgi apparatus. Therefore, it is also important to identify other intracellular molecules acting with MINP as well as the coordinative extracellular factors, both of which may be part of a novel negative regulatory system for radial migration.

MINP contains only one protein domain called the domain of unknown function DUF3585, which is a member of the uncharacterized protein families found in the Pfam database^30^. Interestingly, we found that the C-terminal region of the molecule interacting with CasLs1 (MICAL-1) protein has the same protein domain, which has been proven to be the binding site for plexin^31^. Because MICAL-1 acts as a mediator for sema/plexin signaling, it has been implicated in the regulation of various molecular and cellular processes^31^. However, the effects of MICAL-1 on neuronal migration remain largely uncharacterized. Knowledge of whether MINP and MICAL-1 participate in the same regulatory pathway during neuronal migration may help interpret our results differently.

Hence, our study findings lay the foundation for exploring the function of the novel gene *MINP*. Further analysis for uncovering other distinctive roles of MINP in the nervous system is currently in progress. Because *MINP* is a risk gene located at 16p13.11, transgenic animals carrying *MINP* mutations are expected to serve as new models for neuropsychiatric diseases associated with 16p13.11 CNVs.

## Methods

### Animals

All mice used in experiments were of the slc-ICR strain, and were purchased from SLC Japan, Inc. The mice were bred and maintained at the experimental animal facility of Osaka University Graduate School of Medicine and were killed with an overdose of a mixture of Vetorphale (0.5 mg/mL, Meiji), Dormicum (0.4 mg/mL, Roche) and Domitor (0.03 mg/mL, Orion Pharma) by peritoneal injection. This study was approved by the institutional committee of Osaka University and all experiments were performed in accordance with the Guide for the Care and Use of Laboratory Animals of the Osaka University Medical School.

### Plasmids

pCMV-SPORT6.1 plasmids carrying the full-length cDNA of *MINP* (RIKEN cDNA 2900011O08 gene) were acquired from the Thermo Fisher Scientific K.K. The purified products, tagged with a carboxy-terminal c-Myc epitope, were cloned into a pCAGIG vector (Addgene), producing a pCAG-MINP-Myc-IRES-GFP or pCAG-MINP-Myc expression construct. *MINP* shRNA constructs were generated by cloning and annealing the hairpin sequences to form a DNA insert that contained the hairpin *MINP* siRNA target sequence (MSS289252, Invitrogen). The oligonucleotide sequences were *MINP* shRNA, 5′-GAT CCC CGT GAA GTC TTG GTT ACT TTG TCT AGA TTC AAG AGA TCT AGA CAA AGT AAC CAA GAC TTC ATT TTT A-3′ and 5′-AGC TTA AAA ATG AAG TCT TGG TTA CTT TGT CTA GAT CTC TTG AAT CTA GAC AAA GTA ACC AAG ACT TCA CGG G-3′, scrambled shRNA, 5′-ACT ACC GTT GTT AAG GTG-3′. EF1α promoter driven nuclear EGFP reporter expression plasmid was used as an indication of transfected cells^32^. For rescue experiments, the cDNA of human-*MINP* (C16orf45), which was not targeted by mouse-*MINP* siRNA was amplified and cloned into pCAG-Myc-IRES-GFP vector. The human-*MINP* cDNA was amplified with the following primers: Forward, 5′- TAT CTC GAG CAT GGA GCA GAA GCT GAT CTC AGA GGA GGA CCT GCT TTG GGG CGA CCT CAC AGA G -3′, Reverse 5′-CAG GAC TAT GCG GCC GCC TAC ATG ATG TTG CAC TGG GTG GCC CCG -3′.

### In situ hybridization

DIG-labeled sense and antisense probes were produced by *in vitro* transcription using the full-length (2 kb) cDNA of *MINP*. Brain sections were prepared at 40 μm (adults, 50 μm) and fixed in 4% PFA in 0.1 M PB for 15 min. After washing in DEPC-PBS, the sections were treated with Proteinase K (1 μg/mL, Roche) at 37°C for 30 min followed by fixation with 4% PFA in 0.1 M PB one more time. Hybridization was performed at 70°C overnight and signals were detected using alkaline phosphatase-coupled anti-digoxigenin antibodies (1:5000, Roche). A NBT/BCIP stock solution (1:50, Roche) was used as a substrate for the color reaction. All the solutions used in this experiment were prepared with DEPC-treated water. Images were acquired with a BZ-9000 fluorescence microscope (Keyence).

### RT-PCR analysis

Tissues (cortex, cerebellum, spinal cord, dorsal root ganglia, heart, lung, thymus, salivary gland, skin, muscle, stomach, liver, colon, spleen, pancreas and kidney) from the male or female mice (8 weeks old) were homogenized in Trizol (Invitrogen). RNA was isolated with chloroform and purified using RNeasy Micro Kit (QIAGEN). cDNA synthesis was performed with the High Capacity cDNA Reverse Transcription Kit (Applied Biosystems). For RT-PCR, cDNA was amplified with the following primers: MINP Forward, 5′-ATC CAG CGT CTC CGT GAA GT-3′, Reverse 5′-AGC TGA ATG TCA TCC ATC ATG AAC-3′, Rps18 Forward, 5′-GAT GGG CGG CGG AAA-3′, Reverse 5′-CCC CAC GCC CTT AAT GG-3′.

### Antigen production and antibody purification

*MINP* cDNA was cloned into a pET-29a-N vector (Novagen). After transformation, *Escherichia coli* bacteria were cultured in LB medium (BD) with kanamycin (1:1000, Wako). Growth of the cultures was measured by optical cell density 600 nm (OD600), and IPTG (1:1000, Wako) was added for protein induction. After an additional 3 h of incubation, cultures were lysed by sonication, filtered through Ni-NTA Agarose (QIAGEN) and finally eluted with Imidazole (Sigma). Protein fractions were centrifuged using Amicon ultra-10K (Millipore) and the concentrated MINP antigen was prepared for immunization. Polyclonal anti-MINP antibodies were produced by MBL CO., LTD. The immunized serum was purified with CNBr-activated Sepharose 4B affinity gel (GE imagination) and the antibodies were stored with glycerol at a concentration of 1 μg/μl.

### Immunostaining

For immunohistochemistry, post-fixed E12.5 and E17.5 mouse brains were sectioned at 20 μm. Sections were fixed in 4% PFA in 0.1 M PB for 15 min, followed by blocking and permeabilization with 10% BSA and 0.3% Triton X-100, and finally treated with the M.O.M. blocking kit according to the manufacturer's instructions (Vector Laboratories). For immunocytochemistry, cortical neurons from E14.5–E15.5 mice were cultured in DMEM/F-12 (Gibco) with 2% B27 supplement (Invitrogen). After 2 days *in vitro* (DIV), the neurons were fixed for 10 min in 4% PFA in 0.1 M PB, permeabilized with 0.2% NP-40 for 10 min, and blocked in 6% goat serum and 0.5% BSA in PBS for 1 h. After incubating the sections with primary antibodies overnight at 4°C, secondary antibodies were applied for 1-h incubation at room temperature. Finally, we counterstained with DAPI (1:1000, Dojindo) and sealed the slides with a cover slip and Fluorescence Mounting Medium (Dako). The primary antibodies used for immunolabeling were rabbit anti-MINP (1:800), rabbit anti-GFP (1:800, Invitrogen), mouse anti-Tuj1 (1:1000, Covance), mouse anti-human transferrin receptor (TfnR, 1:250, Invitrogen), mouse anti-syntaxin6 (1:500, BD Transduction Laboratories) and mouse anti-EEA1 (1:250, Santa Cruz). Alexa 488 anti-rabbit IgG (1:500, Invitrogen), Alexa 568 anti-mouse IgG (1:500, Invitrogen) and Cy3-conjugated streptavidin (1:1000, Jackson ImmunoResearch Laboratories) were used as secondary antibodies. Images were acquired using a BX51 fluorescence microscope (Olympus) or FV-1200 laser scanning confocal microscope (Olympus).

### In utero electroporation

In utero electroporations were performed on E14.5 mice using a square wave electroporator CUY21SC (NEPAGENE) that delivered five 50-ms pulses of 40 V with 950-ms intervals as described^32^,^33^. We injected a mixture of a nuclear EGFP expression vector with *MINP*-siRNA (Invitrogen) at a ratio of 1:1. For observing morphology and rescue experiments, 0.375 μg of pCAG-IRES-GFP or pCAG-human-*MINP*-IRES-GFP expression vector mixed with control or *MINP*-siRNA were injected into per embryo at a ratio of 1:5. For shRNA experiments, a total 4.0 μg of plasmids were injected, that consisted of a mixture of nuclear EGFP and *MINP* shRNA expression vectors at a ratio of 1:5, respectively. 0.05% Trypan blue (Gibco) was co-injected as a tracer and control and knock-down brains from the same litter were dissected at E16.5, E17.5 and P2. Mouse anti-Ki67 (1:200, BD Bioscience), mouse anti-HuD (1:200, Molecular Probes), rabbit anti-GFP (1:800, Invitrogen), mouse anti-Satb2 (1:100, Abcam) and rat anti-Ctip2 (1:200, Abcam) were used as primary antibodies. DAPI (1:1000, Dojindo) was used to counterstain nuclei. For neuronal morphology, cells with multipolar and uni-or bipolar morphologies were counted in the intermediate zone, which was previously described^13^. Sections with a similar anatomical distribution of EGFP expression were chosen for analysis, and a total of six to eight sections were analyzed per animal on a BX51 fluorescence microscope (Olympus) or FV-1000 laser scanning confocal microscope (Olympus).

### Cell cultures and transfections

Primary cultures of cortical precursors: Dorsal part of the telencephalon was carefully dissected from E12.5 embryo. The dissected cortices were transferred to the Neurobasal media (Gibco) containing 40 ng/mL FGF2 (Promega), 2% B27 (Gibco), 120 mg/mL penicillin, 200 mg/mL streptomycin sulfate, and 600 mg/mL glutamine for suspension. Cortical precursors were applied at a density of 150,000 cells/well on four-well chamber slides. The cells were maintained in culture media for 1 or 2 days. Cultures were co-incubated at room temperature for 45 min with 1 μg of total plasmid, 1.5 μL Fugene 6.0 (Roche) and 100 μL of Opti-MEM (Invitrogen) for transfection^32^.

Primary cultures of cortical neurons: Cortices from E14.5 mice were digested in 0.25% trypsin (Gibco) with DNase (1:1000, Sigma) for 20 min at 37°C, followed by dissociation in DMEM containing 10% fetal bovine serum (FBS, Invitrogen). Approximately, 5 × 10^6^ cells were nucleofected with control or *MINP* siRNA (60 pM, Invitrogen) as described by the manufacturer (Lonza). Neurons were plated on poly-L-lysine-coated dishes and maintained at 37°C and at 5% CO_2_. Medium was exchanged with DMEM/F-12 (Gibco) containing 2% B27 supplement (Invitrogen) 4 h later. For transfections of HEK293 cells, we used Lipofectamine2000 as outlined by the manufacturer (Invitrogen).

### Western blotting

Cells or cortices were lysed in RIPA buffer (50 mM Tris-HCl, 150 mM NaCl, 1% NP-40, 0.1% SDS, 0.5% sodium deoxycholate) containing protease inhibitors (Roche). Lysates were separated on 12% SDS-polyacrylamide gels and transferred to PVDF membranes. Membranes were blocked with 5% Skim milk in PBS-T (0.05% Tween-20) buffer for 60 min, and incubated with primary antibody at 4°C overnight. The primary antibodies used for immunoblotting were rabbit anti-MINP (1:2000), rabbit anti-ERK1 (1:1000, Santa Cruz), mouse anti-a-tubulin (1:500, Santa Cruz), rat-anti-tyrosinated tubulin (1:1000, Millipore), rabbit anti-de-tyrosinated tubulin (1:1000, Millipore), rabbit-anti-delta 2 tubulin (1:1000, Millipore). After washing with PBS-T, membranes were incubated for 1 h at RT with horseradish peroxidase-conjugated secondary antibodies (1:2000, Cell Signaling Technology). Signals were detected using enhanced chemiluminescence (ECL, GE Healthcare), and images were obtained using a LAS-3000 image analyzer (Fuji Film). Quantification was performed using ImageJ software (NIH).

### Co-immunoprecipitation

E14.5 cortical neurons were nucleofected with pcDNA 3.1 (MOCK) or pCAG-MINP-Myc (MINP) expressing constructs. 48 h later, the cells were harvested and lysed in IP buffer (10 mM Tris pH 7.5, 150 mM NaCl, 1 mM EDTA, 10% glycerol, 0.5% NP40) containing protease inhibitors (Roche). 100 μg cell lysates were immunoprecipitated (IP) with 1 μg mouse anti-c-Myc antibody (Santa Cruz) overnight at 4°C and were immunoblotted (IB) with mouse anti-α-tubulin (1:2000, Santa Cruz) and mouse anti-c-Myc (1:2000, Santa Cruz). Immunoprecipitation without anti-Myc antibody was used as negative control.

### Cell migration assay

E14.5 cortical neurons were transfected with control or *MINP*-siRNA. 2 days after transfections, cells were re-plated on the insert of the Boyden chamber (3 μm pore size, Corning Costar) at a concentration of 5 × 10^4^ cells. The insert was placed in the lower chamber which contained DMEM/F-12 with 2% B27 supplement. As a positive control, 50 ng/mL brain-derived neurotrophic factor (BDNF; Millipore) was added to the lower chamber. DAPI (Santa Cruz) staining was performed on the bottom side of the insert 16 h later. For quantification, cells from 10 randomly chosen fields on the bottom side of the insert were counted. Images were acquired with BX51 fluorescence microscope (Olympus).

### Tubulin assay

The assay was performed as described previously^20^. E14.5 cortical neurons transfected with control or *MINP*-siRNA were harvested after 3 DIV. As positive control, the mitotic inhibitor Taxol (50 nM, Sigma) was added to the medium 4 h before harvesting.

### Statistics

Data from the *in vitro* cell migration and tubulin assays were compared using one-way ANOVAs, followed by Tukey–Kramer tests. Results from *in vivo and in vitro* proliferation assays, the differentiation assays and the *in vivo* migration assays were analyzed using Student's *t* tests. For histograms of immunoblots, differences were compared using Student's *t* tests following normalization to the control. All data were presented as mean ± SEM.

## Supplementary Material

Supplementary Information supplementary
