# Supplementary material for: The newly identified migration inhibitory protein regulates the radial migration in the developing neocortex
Source: Sci Rep. 2014 Aug 7;4:5984. doi: 10.1038/srep05984 (PMC5380009; doi:10.1038/srep05984)

Supplementary Information

THE NEWLY IDENTIFIED MIGRATION INHIBITORY PROTEIN REGULATES THE RADIAL MIGRATION IN THE DEVELOPING NEOCORTEX

Suxiang Zhang[1,2], Yoshitaka Kanemitsu[1,2], Masashi Fujitani[1,2*], Toshihide Yamashita[1,2*]

1Department of Molecular Neuroscience, Graduate School of Medicine, Osaka University,
2-2 Yamadaoka, Suita, Osaka 565-0871, Japan
2JST, CREST, 5, Sanbancho, Chiyoda-ku, Tokyo 102-0075, Japan

* Address correspondence to: Masashi Fujitani and Toshihide Yamashita
Department of Molecular Neuroscience, Graduate School of Medicine, Osaka University,
2-2 Yamadaoka, Suita, Osaka 565-0871, Japan

Supplementary Figure 1

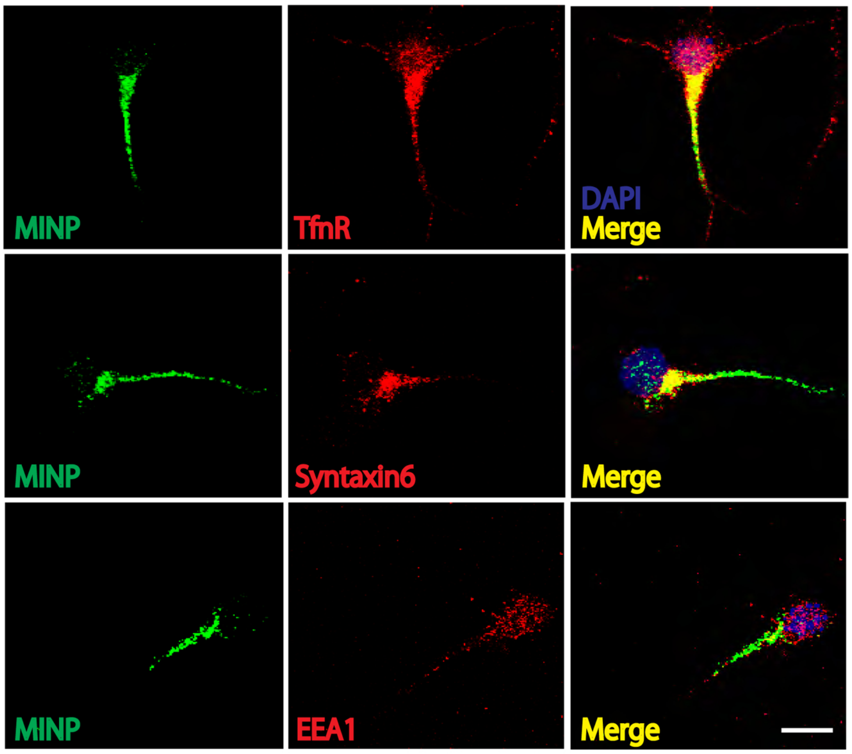

Supplementary figure 1.
Subcellular localization of MINP. E14.5 cortical neurons were fixed after 2 days of culture and double stained for MINP and indicated several subcellular compartments. Confocal micrographs show that MINP was partially overlapped with TfnR (a marker for recycling endosomes) and Syntaxin6 (a marker for trans-Golgi network) but was not co-localized with EEA1 (a marker for early endosomes).
Scale bar, 10 μm.

Supplementary Figure 2

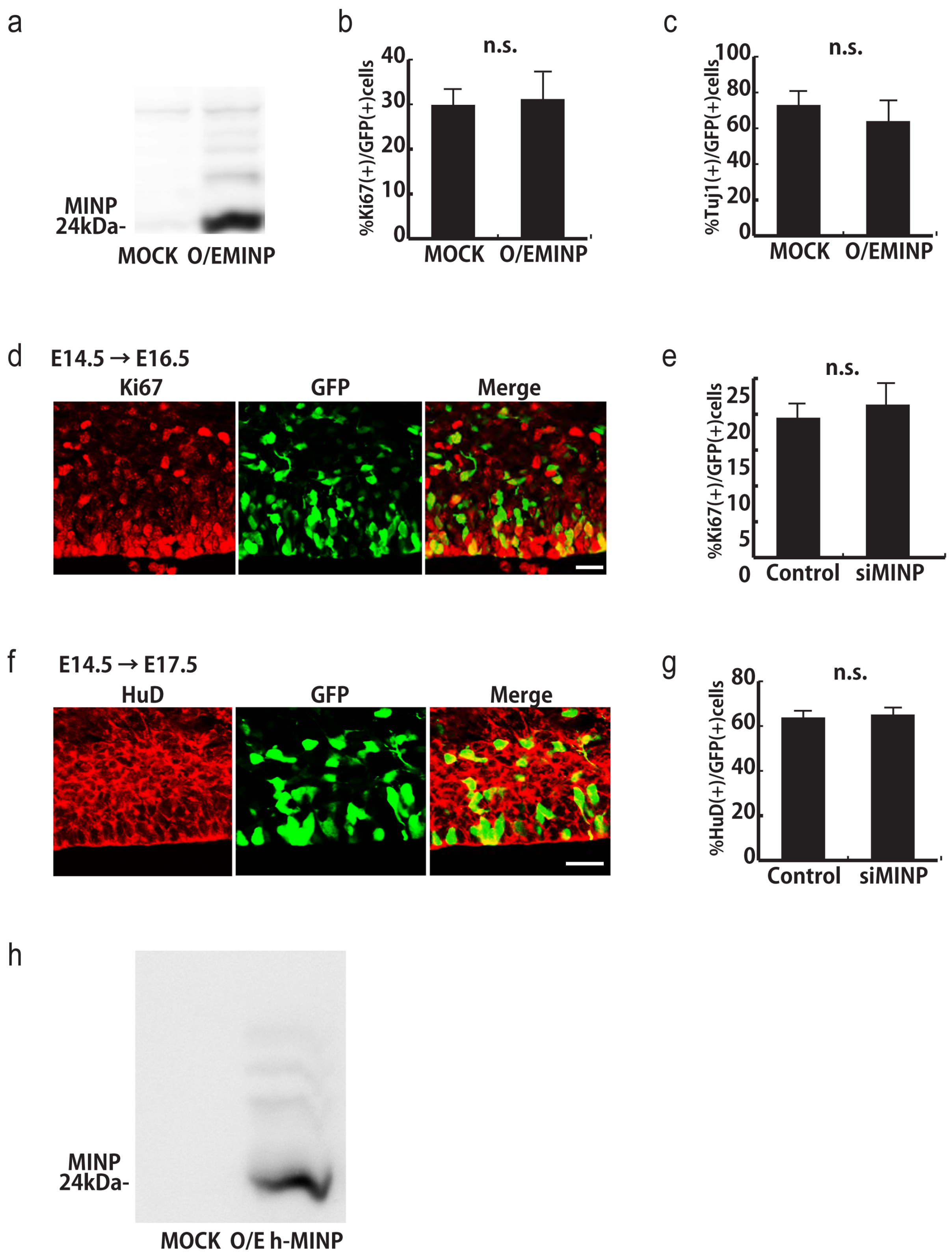

Supplementary figure 2.

Overexpression or knockdown of MINP does not affect proliferation or differentiation of cortical precursor cells. (a) Transfections of control (MOCK) or MINP-overexpressing vector (O/EMINP) in HEK293 cells. The overexpression efficiency was assessed at 2 days after transfection. (b-c) E12.5 cortical precursors were transfected with control or MINP overexpressing vector, followed by immunostaining for Ki67 at 2 days or Tuj1 at 3 days. Quantification of the transfected cells that positive for Ki67 (b) or Tuj1 (c) (mean ± SEM; n = 3, each group). (d-g) E14.5 cortices were electroporated with control or MINP siRNA. Nuclear GFP was co-transfected as a tracer. Sections were obtained at E16.5 and E17.5, followed by immunostaining for the proliferation marker Ki67 (d) or the neuronal marker HuD (f). Scale bar, 20 μm. Quantification shows that knockdown of MINP does not affect proliferation (e) or differentiation (g) of cortical precursor cells (mean ± SEM; n = 3, each group). (h) Transfection of control (MOCK) or human-MINP-Myc overexpressing vector (O/E h-MINP) in HEK293 cells. The overexpression efficiency was assessed at 2 days after transfection by immunoblotting with anti-Myc antibody.

Supplementary Figure 3

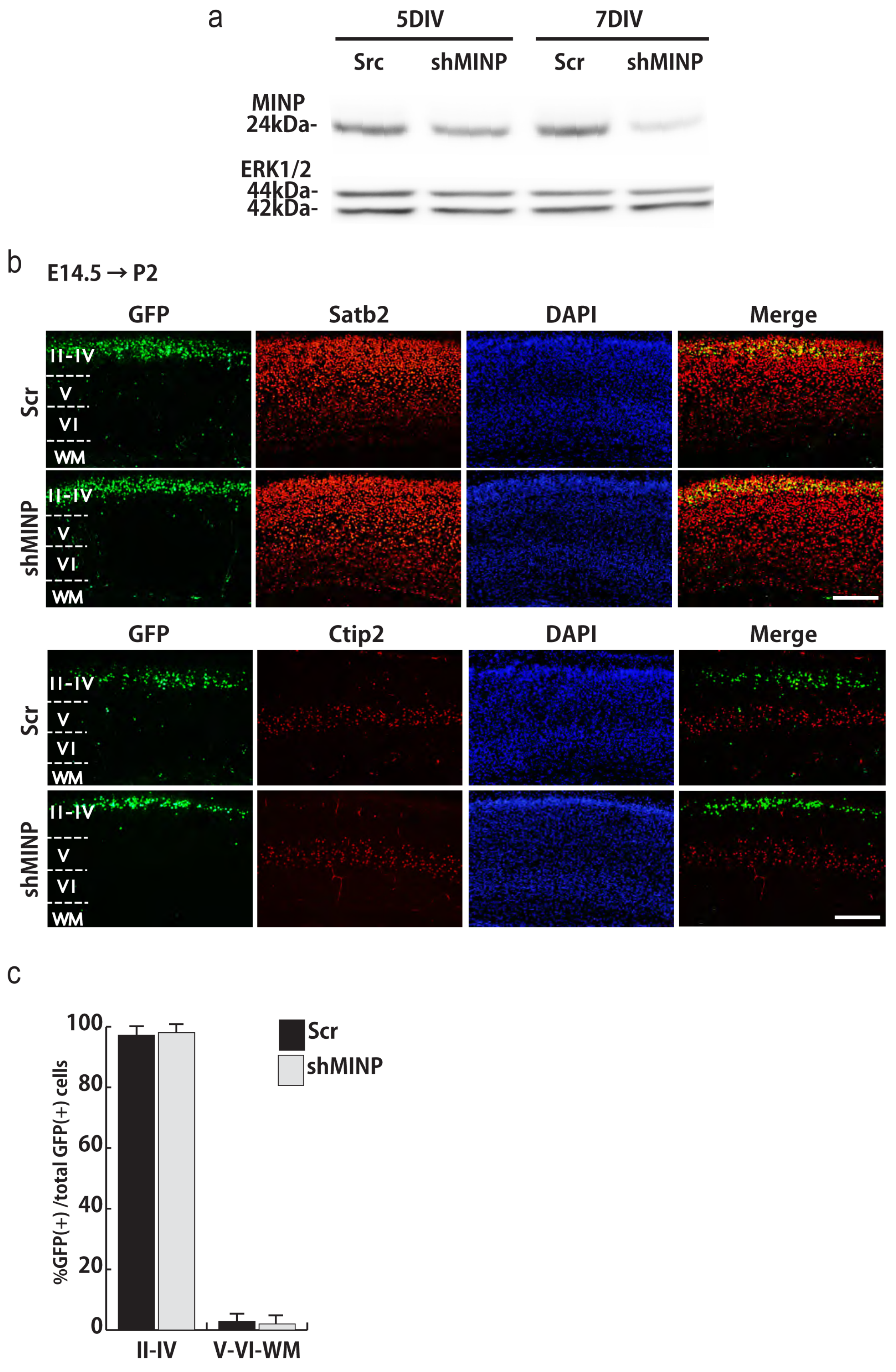

Supplementary figure 3.

Final destination of neurons is not affected by longer knockdown of MINP. (a) Western blots used for quantifying the knockdown efficiency of MINP-shRNA vectors in E14.5 cortical neurons at the indicated days in vitro. ERK1/2 was used as a loading control. (b) Scrambled (Scr) or MINP shRNA (shMINP) was electroporated in combination with nGFP in E14.5 cortices. Sections were analyzed at P2, following immunostaining for nGFP (green), Satb2 (red, upper two panels) or Ctip2 (red, lower two panels) in combination with DAPI (blue). WM, white matter. Scale bar, 100 µm. (c) Quantification of the layer distribution of GFP positive cells shows no significant difference between the scrambled and MINP shRNA-electroporated cortices (mean ± SEM; n = 3, each group).

Figure 1

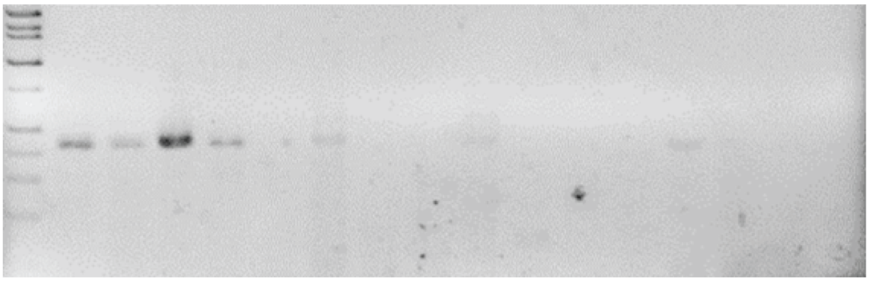

Figure 2

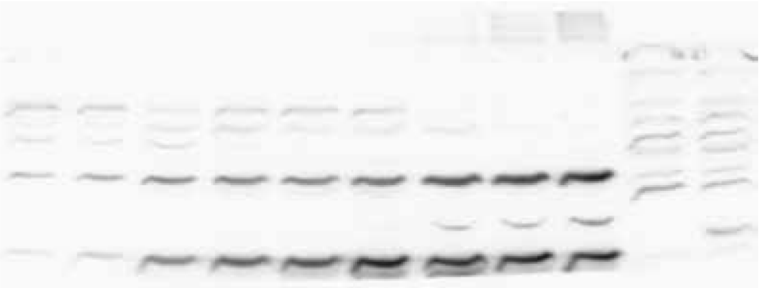

Figure 3

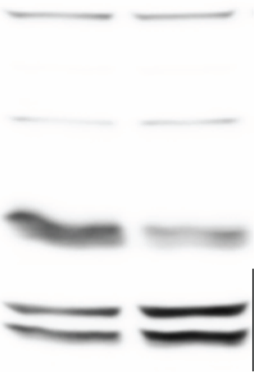

Figure 6

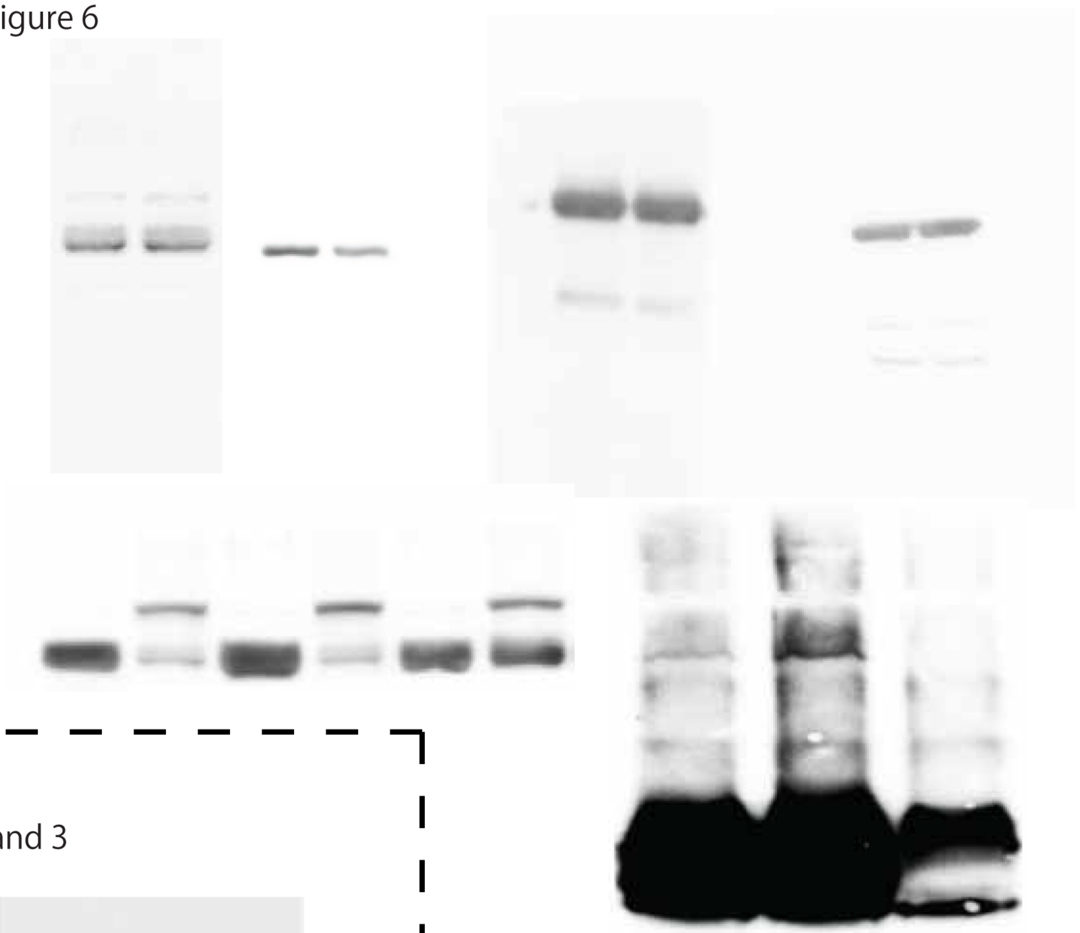

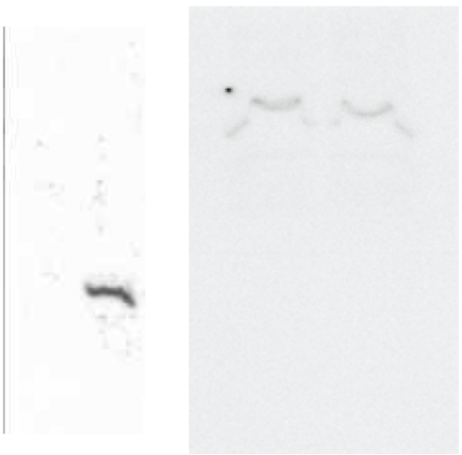

Supplementary figure 2 and 3

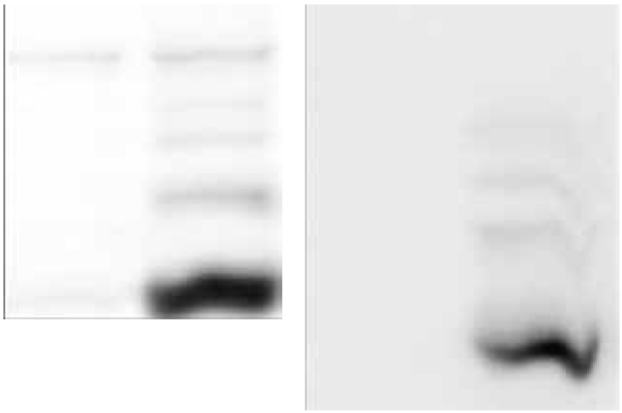

Supplement: Supplementary Information — supplementary [file srep05984-s1.pdf]
